# A case of intramuscular myxoma presenting as a swollen shoulder: a case report

**DOI:** 10.1186/1752-1947-8-441

**Published:** 2014-12-18

**Authors:** Bahattin Kemah, Mehmet Salih Soylemez, Bahar Ceyran, Serkan Şenol, Serhat Mutlu, Korhan Özkan

**Affiliations:** 43 Orthopaedics and Traumatology Department, İ. Medeniyet University Göztepe Training and Research Hospital, İstanbul, Turkey; 44 Pathology Department, İ. Medeniyet University Göztepe Training and Research Hospital, İstanbul, Turkey; 45 Orthopaedics and Traumatology Department, İ. Kanuni Sultan Suleyman Training and Research Hospital, İstanbul, Turkey; 46 Orthopaedics and Traumatology Department, İ. Medeniyet University, İstanbul, Turkey

**Keywords:** Benign tumor, Deltoid muscle, Intramuscular myxoma, Myxoma

## Abstract

**Introduction:**

Intramuscular myxoma is a rare benign mesenchymal tumor. Myxomas most commonly occur in the heart. They may occur less frequently in aponeurotic tissues, bone, genitourinary tract, subcutaneous tissue and skin.

**Case presentation:**

The case described here is a 44-year-old Turkish woman who presented with the complaint of a swelling in her right shoulder. A preoperative magnetic resonance imaging revealed a lobular contoured mass lesion in her deltoid muscle. The mass was marginally excised and pathology revealed intramuscular myxoma.

**Conclusion:**

Intramuscular myxoma of the deltoid muscle is a very rare benign tumor. In the differential diagnosis, reactive lesions, myxoid nodular fasciitis and low -grade myxoid sarcomas should be kept in mind, upon which the treatment should be planned.

## Introduction

Myxomas have been described as a true neoplasm of low-vascularity composed of undifferentiated satellite cells embedded in myxoid stroma containing collagen and reticular fibers [1]. Myxoma is the most common intracardiac (atrial) tumor [2]. Extracardiac myxomas may occur in aponeurotic tissues, bone, genitourinary tract, skin, retroperitoneum, intestine, pharynx, joints and skeletal muscles [1]. Osseous myxomas have been reported in the jawbones and calcaneum [3]. Extracardiac myxomas are benign tumors with a slow growth rate and low mitotic activity, which are surrounded by skeletal muscles; they usually present in the 4th to 6th decades of life in women [4–7].

Intramuscular myxoma usually occurs as an isolated lesion. The first case of intramuscular myxoma was described in 1965 [8]. Intramuscular myxoma rarely occurs as multiple lesions associated with fibrous dysplasia of the bone (Mazabraud syndrome) or as a part of McCune–Albright syndrome [2, 9–13].

Here we report a case of a 44-year-old woman with myxoma located in her right deltoid muscle and aim to discuss intramuscular myxomas in the light of clinical, radiological and pathological data.

## Case presentation

A 44-year-old Turkish woman presented to our clinic with the complaint of a 3-year history of a slow growing palpable mass in her right shoulder. She had no other documented or reported condition.

Her physical examination revealed a palpable, mobile, painless, partially fluctuating mass, 5×5cm in size, in her deltoid muscle, extending to the lower lateral part of her right shoulder and proximal lateral part of her right arm.

**Figure 1:**
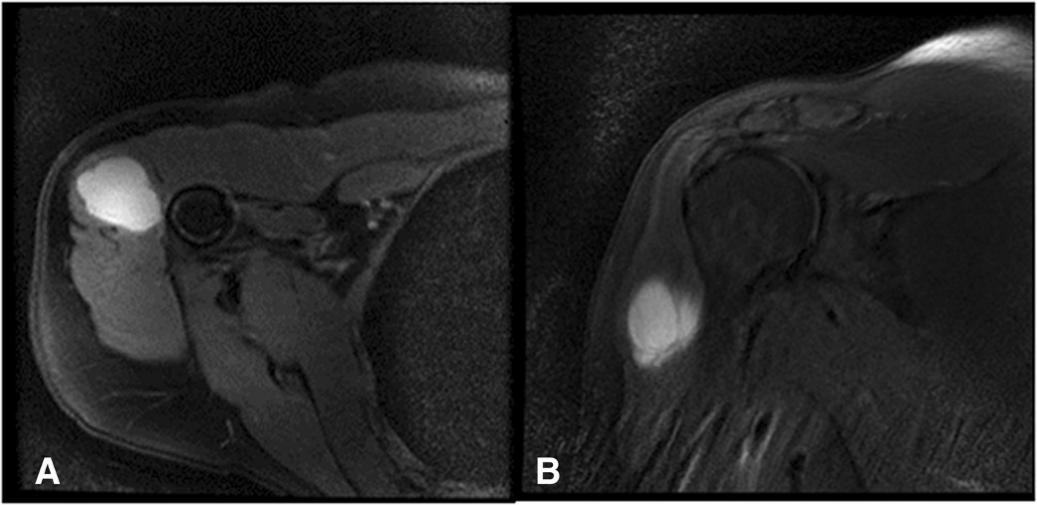
**Appearance of the mass on T2-weighted magnetic resonance imaging sections. A)** In axial section; **B)** in coronal section.

Magnetic resonance imaging (MRI) revealed a lobular contoured mass lesion, cystic in nature and measuring 34×23×37mm at its largest that was located caudal to the lateral compartment of her deltoid muscle and an interstitial edema in the adjacent deltoid muscle (Figure 1). A complete blood count, biochemical and other laboratory tests revealed normal values.

During her surgical operation, the mass was approached via a longitudinal incision over the mass in the deltoid area of her right shoulder, sensory neural branches of the axillary nerve were preserved, and the encapsulated gelatinous mass 4×5cm in size was easily separated from the muscle tissue and then excised. A macroscopic pathological examination of the mass revealed a nodular lesion cream-beige in color, 2.5×2.5×3cm in size and capsular in appearance that was adjacent to the deltoid muscle. The sectioned surface of the mass was pink-beige in color and solid bright myxoid in appearance. The surgical margin was tumor-free.

**Figure 2:**
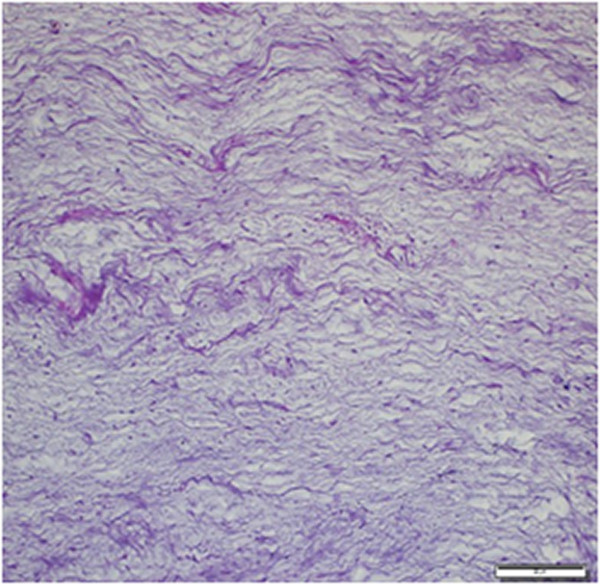
**Tumor lesion with a small number of spindle or stellate cells, a small number of vessels, and abundant thin collagen fibers (hematoxylin and eosin ×100).**

**Figure 3:**
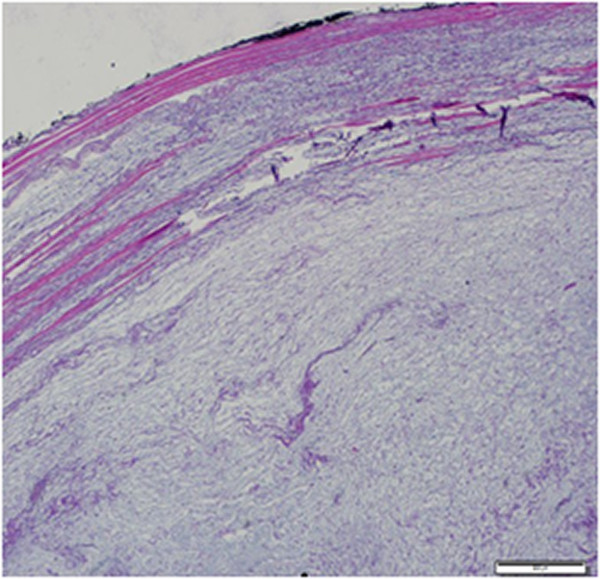
**At the periphery of the lesion, the skeletal muscle adjacent to the tumor is atrophic.** Tumor infiltration of muscle fibers can be monitored (hematoxylin and eosin ×200).

**Figure 4:**
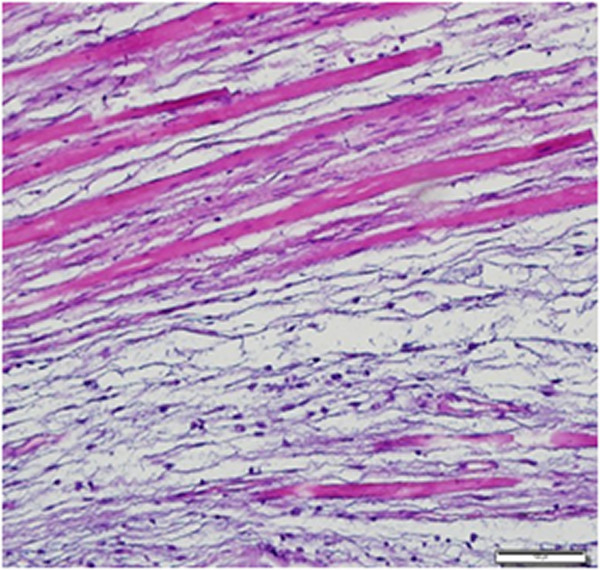
**Appearance of the infiltrating tumor with atrophic surrounding muscle fibers at higher magnification.**

A microscopic examination showed that the mass consisted of a small number of spindle or stellate cells, a small number of vessels, thin collagen fibers and a small number of loose reticular fibers embedded in an abundant myxoid stroma (Figure 2). The cells had a small, pyknotic slightly hyperchromatic nucleus and scanty cytoplasm. Some presented multiple stellate-shaped cytoplasmic extensions. Cellular pleomorphism, cellularity and vascularity were low. There was no mitosis, necrosis or cystic degeneration. At the periphery of the lesion, the skeletal muscle adjacent to the tumor was atrophic with interspersed edema fluid or infiltrated tumor cells (Figures 3 and 4).

**Figure 5:**
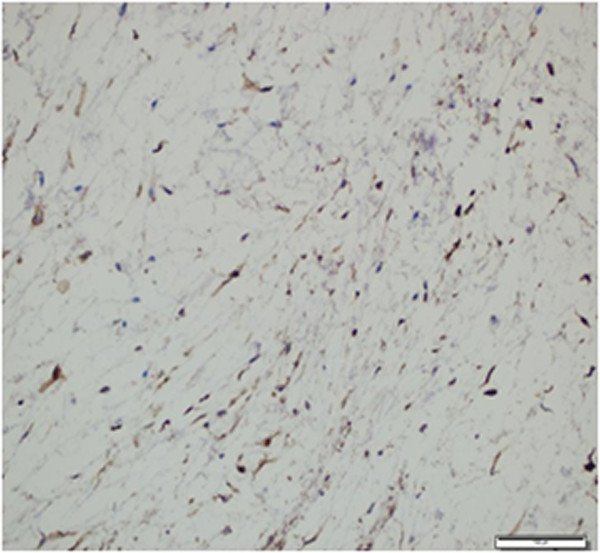
**Immunohistochemical positivity in tumor cells (vimentin ×200).**

**Figure 6:**
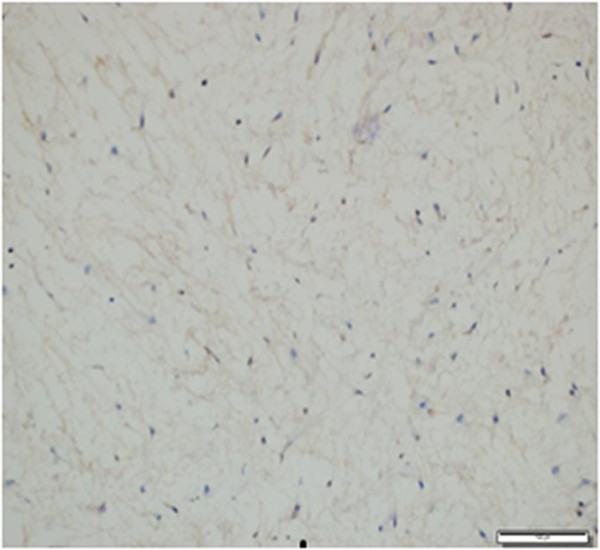
**Immunohistochemical Ki-67 proliferation index is less than 1% in tumor cells (Ki-67 ×200).**

An immunohistochemical examination of the mass showed diffuse positivity for vimentin in tumor cells (Figure 5), focal and weak positivity for CD34 in peripheral regions and was negative for S-100. The Ki-67 proliferation index was less than 1%. In the light of pathological data obtained, the mass was concluded to be intramuscular myxoma (Figure 6).

## Discussion

Myxoma is a benign soft tissue tumor of unknown origin [1]. It may originate from fibroblasts that are insufficiently differentiated and thus unable to synthesize collagen or it may originate from mesenchymal stem cells [9, 14]. It was first described by Virchow in 1863 as a benign tumor similar to mucous tissue of the umbilical cord [15]. It was described as a mesenchymal neoplasm composed of undifferentiated stellate cells embedded in myxoid stroma containing weak reticular fibers in 1948 by Stout [1]. The etiology of myxomas remains elusive. Some authors suggest that the etiology of myxomas may be traumatic [16]. One of the theories on their etiology is the growth of polysaccharide-producing cells in the neoplastic process [8, 16, 17].

Soft tissue myxomas are rare [1]. Extracardiac myxomas most commonly occur in the head and skin tissue. Enzinger, in 1965, identified characteristic properties of a myxoma removed from intramuscular tissue, and described it as intramuscular myxoma [8].

Intramuscular myxomas can occur in the muscles of the thigh, buttocks, shoulder and upper extremities [9]. Intramuscular myxomas primarily affect patients between 40 and 70 years of age, with female predominance [4–7]. They usually follow an asymptomatic course and the most common clinical finding is a slow growing mass [4, 6–8, 16, 18]. In the presence of an association of multiple intramuscular myxomas with fibrous dysplasia, Mazabraud syndrome (multiple intramuscular myxomas and fibrous dysplasia) or McCune–Albright syndrome (multiple intramuscular myxomas and polyostotic fibrous dysplasia, café-au-lait stains, endocrine hyperfunction) should be considered [2, 9–14, 19–22]. The case presented here had a single myxoma with no bone pathology.

On clinical examination, intramuscular myxoma presents as a painless, palpable mass. The clinical pattern varies depending on the size and site of the mass [14, 23].

A radiograph reveals a nonspecific mass with no calcification. On ultrasonography, it appears as cystic lesions with echogenicity in normal muscle tissue [24]. On computed tomography, it has a homogenous appearance separating from the muscle tissue, similar to that of a cyst or low density mass [25]. The most important radiologic examination used for the mass is MRI. Intramuscular myxoma appears as a hypointense homogeneous mass on T1-weighted sections, and as a hyperintense mass on T2-weighted sections [2, 25–29]. Fat-suppressed T2 sections demonstrate increased signal intensity associated with the increased water content of myxoid matrix [25]. Increased intensity can be detected around the mass resembling fat on T1-weighted sections. On T2-weighted sections, edema is observed in the surrounding muscle tissue [2, 25, 26]. These MRI findings may be confused with other fluid-containing lesions such as myxoid sarcoma, hematoma, lymph nodes, cystic hygroma, cystic teratoma and abscess. The case presented here had a lobular contoured mass lesion, cystic in nature in the deltoid muscle. There was interstitial edema in the surrounding tissue. Other possible lesions were borne in mind in the differential diagnosis.

The differential diagnosis of intramuscular myxoma includes other myxoid neoplasms and proliferative lesions of the soft tissue [6]. Benign lesions such as myxolipoma, myxoid neurofibroma, neurothecoma, myxochondroma and nodular fasciitis and different types of low grade myxoid sarcomas should also be considered. Among them, the differential diagnosis should be established with low grade myxofibrosarcoma, myxoid liposarcoma, extraskeletal myxoid chondrosarcoma and low grade fibromyxoid sarcomas [6, 9, 14].

Initial diagnosis can be checked by needle biopsy [7, 24] whereas several authors use intraoperative frozen section [30]. Differential diagnosis with benign lesions can be easily made by histological examination whereas it is difficult to make differential diagnosis by imaging techniques due to myxoid stroma inside myxomas [31]. In our case, an excisional biopsy was planned and the mass was excised at the surgical margin and a histopathological examination was performed.

There are transition zones between hypocellular myxoid areas and fat tissues in myxolipoma. Myxoid liposarcoma includes atypical lipoblasts and characteristic diffuse plexiform capillary network. Myxomas do not contain lipoblasts and have poor vascularity. Myxolipomas and liposarcomas stain positive for S-100 [19].

Neurofibroma cells display a greater degree of orientation whereas the vascularity is more prominent and they contain abundant collagen fibers. Some areas show positive staining for S-100. Neurothecomas contain prominent lobules separated by fibrous bands. Lobules consist of myxoid and cellular areas and stain positive for S-100 [19].

Myxochondromas contain lobular patterns with more cellular chondroid cell groups among myxoid areas, which usually stain positive for S-100. Myxoid chondrosarcomas present more pleomorphic, cellular and infiltrative growth [19].

Nodular fasciitis stains positive for smooth muscle. In myxomas, there is zonal organization or regional heterogeneity of nodular fasciitis. Myxomas are more hypocellular and have poor vascularity [19].

Low grade myxofibrosarcomas differ from myxomas in being more pleomorphic and ill defined, and in having a characteristic nodular pattern and organized vascular pattern. Low grade fibromyxoid sarcomas are characterized by contrasting myxoid and cellular fibrous areas with a swirling, whorled pattern [19].

Treatment of solitary myxomas is marginal surgical excision [7, 9, 32]. Extensive surgical resection is recommended by some surgeons [23]. No metastasis, recurrence or malignant change has been reported with this surgical procedure. However, recurrence has been reported in a small number of patients undergoing enucleation and incomplete resection [6, 33]. Recurrent myxoma with atypical localization has been reported in patients with the association of Mazabraud syndrome and McCune–Albright syndrome [2, 11, 34, 35].

In our case, no needle biopsy was performed and the entire mass was removed with wide margins along with the surrounding muscle tissue and the surgical specimen was sent for pathological examination. Histopathological examination revealed intramuscular myxoma. The patient’s treatment was completed with extensive surgical excision in one session. Currently, she is at the postoperative 6-month follow-up with no complaints or recurrence.

## Conclusions

In conclusion, intramuscular myxoma of the deltoid muscle is a very rare benign tumor. Even though imaging techniques are helpful, the exact diagnosis is established by histological examination. In the differential diagnosis, reactive lesions, myxoid nodular fasciitis and low grade myxoid sarcomas should be kept in mind, upon which the treatment should be planned.

## Consent

Written informed consent was obtained from the patient for publication of this case report and accompanying images. A copy of the written consent is available for review by the Editor-in-Chief of this journal.

## Abbreviations

MRI: Magnetic resonance imaging.
